# Use of coronary physiology to guide revascularization in clinical practice: results of the F(FR)^2^ registry

**DOI:** 10.1007/s00392-024-02463-w

**Published:** 2024-06-04

**Authors:** J. Michael Altstidl, Stephan Achenbach, Johannes Feyrer, J. Benedikt Nazli, Mohamed Marwan, Luise Gaede, Helge Möllmann, Tom Giesler, Harald Rittger, Matthias Pauschinger, Tanja K. Rudolph, Werner Moshage, Martin Brück, Monique Tröbs

**Affiliations:** 1 Department of Medicine 2 – Cardiology and Angiology, Friedrich-Alexander-Universität Erlangen-Nürnberg, Ulmenweg 18, 91054 Erlangen, Germany; 2 Department of Medicine 1 – Cardiology, Nephrology, Intensive Care and Rhythmology, St. Johannes Hospital Dortmund, Dortmund, Germany; 3 Department of Cardiology, Helios Klinik Jerichower Land, Burg, Germany; 4 Department of Cardiology and Pulmonology, Hospital Fürth, Fürth, Germany; 5 Department of Medicine 8 – Cardiology, Nuremberg Hospital South, Nuremberg, Germany; 6 Department of General and Interventional Cardiology, Heart and Diabetes Center NRW, Bad Oeynhausen, Germany; 7 Department of Cardiology, Hospital Traunstein, Traunstein, Germany; 8 Department of Medicine 1, Hospital Wetzlar, Wetzlar, Germany

**Keywords:** Coronary artery disease, Coronary physiology, Fractional flow reserve (FFR), Complications, Registry

## Abstract

**Background:**

Despite the recommendation of coronary physiology to guide revascularization in angiographically intermediate stenoses without established correlation to ischemia, its uptake in clinical practice is slow.

**Aims:**

This study aimed to analyze the use of coronary physiology in clinical practice.

**Methods:**

Based on a multicenter registry (Fractional Flow Reserve Fax Registry, F(FR)^2^, ClinicalTrials.gov identifier NCT03055910), clinical use, consequences, and complications of coronary physiology were systematically analyzed.

**Results:**

F(FR)^2^ enrolled 2,000 patients with 3,378 intracoronary pressure measurements. Most measurements (96.8%) were performed in angiographically intermediate stenoses. Out of 3,238 lesions in which coronary physiology was used to guide revascularization, revascularization was deferred in 2,643 (78.2%) cases.

Fractional flow reserve (FFR) was the most common pressure index used (87.6%), with hyperemia induced by an intracoronary bolus of adenosine in 2,556 lesions (86.4%) and intravenous adenosine used for 384 measurements (13.0%). The route of adenosine administration did not influence FFR results (change-in-estimate -3.1% for regression model predicting FFR from diameter stenosis). Agreement with the subsequent revascularization decision was 93.4% for intravenous and 95.0% for intracoronary adenosine (p = 0.261).

Coronary artery occlusion caused by the pressure wire was reported in two cases (0.1%) and dissection in three cases (0.2%), which was fatal once (0.1%).

**Conclusions:**

In clinical practice, intracoronary pressure measurements are mostly used to guide revascularization decisions in angiographically intermediate stenoses. Intracoronary and intravenous administration of adenosine seem equally suited. While the rate of serious complications of wire-based intracoronary pressure measurements in clinical practice seems to be low, it is not negligible.

**Graphical abstract:**

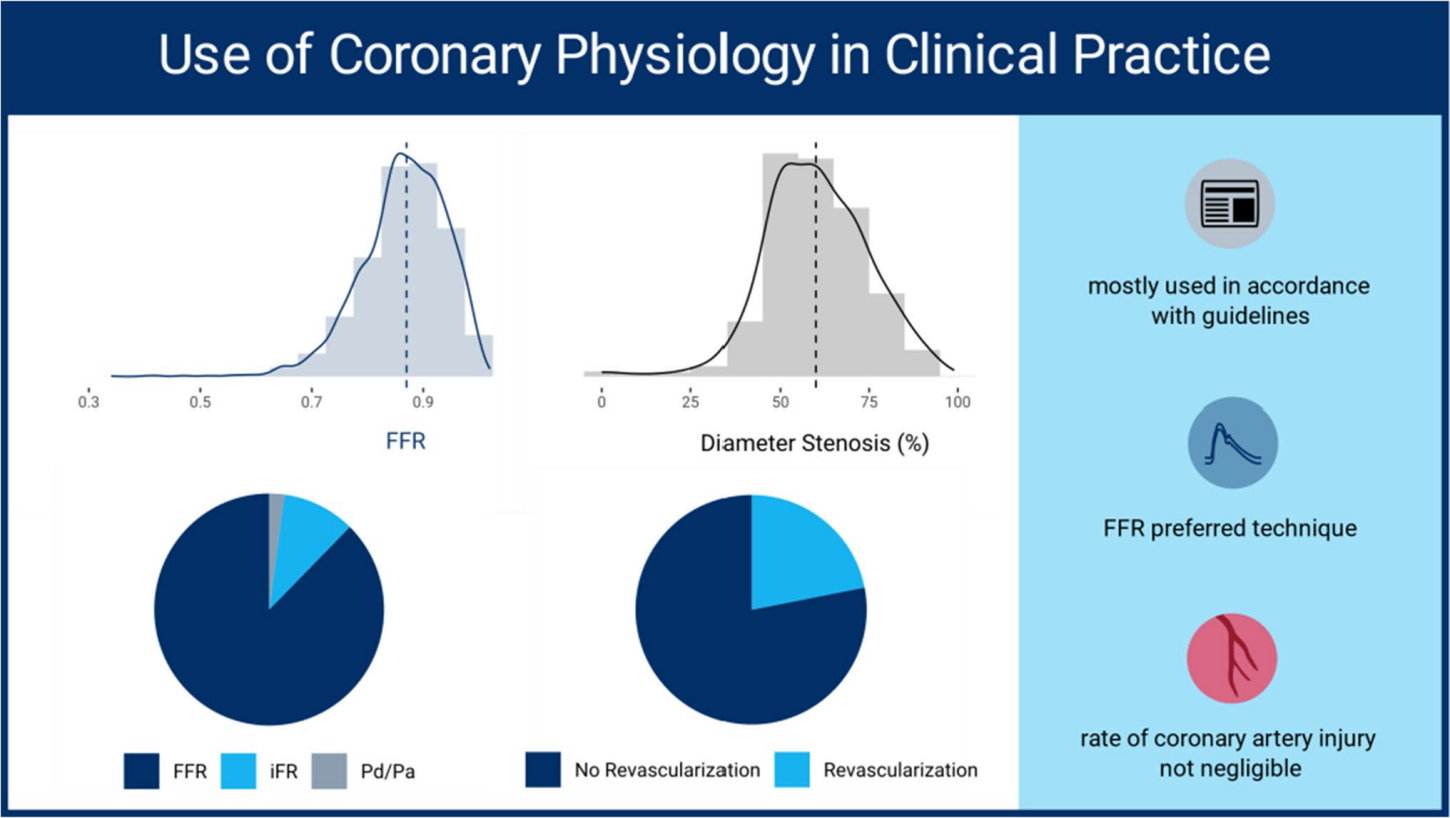

## Introduction

In angiographically intermediate coronary stenoses, current guidelines recommend the use of coronary physiology, particularly fractional flow reserve (FFR) and instantaneous wave-free ratio (iFR), to guide revascularization decisions [1–3]. In numerous trials, FFR-guided revascularization has been shown to significantly reduce the rate of major adverse cardiac events as compared to a purely angiography-guided revascularization approach [4, 5]. Two large randomized controlled trials initially demonstrated noninferiority of iFR to FFR [6, 7], so that the adenosine-independent pressure index is currently equally recommended for the physiological assessment of coronary lesions [1–3]. Of note, the pooled 5-year outcome data of the same trials suggest inferiority of iFR over FFR regarding all-cause mortality [8, 9], but the interpretation of this data in a clinical context remains debated [9].

While numerous registries have focused on the clinical outcomes of revascularization guided by coronary physiology [10–13], its relatively slow uptake in clinical practice in spite of convincing scientific evidence and strong guideline recommendations is not well understood. Data indicate that in clinical practice, pressure indices are only measured occasionally in Germany [14] and elsewhere in the world [15, 16].

Concerns about complications and side effects associated with the intracoronary pressure measurement are assumed to contribute to its relatively infrequent use in clinical practice [17]. In addition, the continuous intravenous infusion of adenosine for FFR measurement may be regarded as time consuming and cumbersome [17]. Hence, given its comparable diagnostic accuracy [18], intracoronary administration of adenosine may provide a convenient and more readily acceptable alternative to induce hyperemia for FFR measurement.

Therefore, this study aimed to analyze the use of intracoronary pressure index measurements and subsequent revascularization decisions in clinical practice as well as its associated complications. It furthermore aimed to identify systematic differences between an intravenous and intracoronary administration of adenosine for FFR measurement in order to investigate whether intracoronary bolus administration of adenosine is equivalent to intravenous adenosine regarding their impact on the revascularization decision in a large, multicenter registry.

## Methods

### Study design

The Fractional Flow Reserve Fax Registry (F(FR)^2^) was an investigator-initiated, multicenter registry which prospectively enrolled 2,000 consecutive patients ≥ 18 years in whom at least one invasive pressure index measurement was attempted for clinical reasons at 8 experienced interventional cardiology centers in Germany (ClinicalTrials.gov identifier NCT03055910). All decisions regarding pressure index measurement and subsequent revascularization were left to the interventionalists´ discretion. Patient as well as procedural and technical characteristics were prospectively collected at the time of inclusion.

This study was performed in accordance with the ethical standards as laid down in the 1964 Declaration of Helsinki and its later amendments. The study protocol was approved by the institutional review board or ethical committee of each participating center. All patients provided written informed consent prior to enrollment.

### Statistical analysis

Statistical analysis was performed with R and its *table1* and *tidyverse* libraries [19–21]. Continuous variables are expressed as medians and interquartile ranges (IQR), while categorial characteristics are expressed as absolute values and percentages. Data were compared with the Wilcoxon rank sum test with continuity correction for continuous variables and with the chi-squared test for categorical variables. Distributions were compared with the two-sample Kolmogorov–Smirnov test. For all analyses, a p value < 0.05 was considered to indicate statistical significance.

## Results

### Patient characteristics

Over a period of 51 months, 2,000 patients were enrolled. Patient characteristics are summarized in Table 1. The median age was 69 (IQR: 60–76) years. With only 544 (27.2%) of the enrolled patients being female, women were underrepresented in this registry. Coronary angiography was performed due to chronic coronary syndrome (CCS) in most patients, i.e., 1,556 (77.8%) patients, compared to 300 (15.0%) patients with acute coronary syndrome (ACS) and 144 (7.2%) patients with other indications such as valvular heart disease. About one-half of all patients (*n* = 996, 49.8%) had undergone previous revascularization, including previous percutaneous coronary intervention (PCI) in 945 patients (47.3%) and coronary artery bypass graft surgery (CABG) in 89 (4.5%).

**Table 1:** Patients and procedural characteristics (*n* = 2,000 patients)

Age, years	69 [60–76]
Sex, male	1,456 (72.8%)
Indication for angiography and clinical presentation	
STEMI	9 (0.5%)
NSTEMI	94 (4.7%)
Unstable Angina	197 (9.9%)
CCS	1,556 (77.8%)
Other	144 (7.2%)
Previous Revascularization	996 (49.8%)
PCI	945 (47.3%)
CABG	89 (4.5%)
Median number of pressure index measurements per patient	1 [1-2]
Aspirin	534 (26.7%)
Heparin	1,974 (98.7%)
Nitrate	1,595 (79.8%)
Pressure Wire / Microcatheter	
PressureWire™ X (Abbott)	1,553 (76.5%)
Verrata® PLUS (Philips)	454 (22.4%)
Navvus® (ACIST)	23 (1.1%)

Values are median [IQR] or n (%). CABG indicates coronary artery bypass graft; *CCS* chronic coronary syndrome, *NSTEMI* non–ST-segment–elevation myocardial infarction, *PCI* percutaneous coronary intervention, and *STEMI* ST-segment–elevation myocardial infarction

### Procedural and technical characteristics

A total of 3,378 invasive pressure index measurements were performed in the 2,000 patients, with a median of 1 (IQR: 1–2) invasive pressure index measurements per individual. Procedural and technical characteristics are summarized in Tables 1 and 2. In the context of coronary angiography and intracoronary pressure measurements, 26.7% of patients received aspirin, 98.7% of patients received heparin, and 79.8% of patients received intracoronary nitrates. Pressure index measurements were performed using a total of 2,030 pressure wires or pressure-monitoring microcatheters, more specifically 1,553 PressureWire™ X pressure guide wires (Abbott Laboratories, Abbott Park, Illinois), 454 Verrata® PLUS pressure guide wires (Philips, Amsterdam, The Netherlands) and 23 Navvus® pressure-monitoring microcatheters (ACIST Medical Systems, Inc, Eden Prairie, Minnesota).

**Table 2:** Procedural and Technical Characteristics (*n* = 3,378 lesions)

Lesion location	
LM	71 (2.1%)
LAD	1,964 (58.1%)
LCX	745 (22.1%)
RCA	598 (17.7%)
Side branch	414 (12.3%)
Bypass graft	10 (0.3%)
Culprit lesion	125 (3.7%)
Pressure index	
FFR	2,960 (87.6%)
iFR	346 (10.2%)
P_d_/P_a_	72 (2.1%)
Hyperemic agent	
Intravenous adenosine	384 (13.0%)
Intracoronary adenosine	2,556 (86.4%)
Intravenous regadenoson	10 (0.3%)
Pullback	198 (5.9%)
Visually estimated diameter stenosis, %	60 [50–70]
< 40%	95 (2.8%)
40–90%	3,269 (96.8%)
> 90%	14 (0.4%)
Pressure index measurement result	
FFR	0.87 [0.34–1.02]
iFR	0.94 [0.36–1.12]
P_d_/P_a_	0.90 [0.66–1.04]
Timing and indication	
Before PCI (to guide revascularization)	3,238 (95.9%)
After PCI (to evaluate result)	140 (4.1%)
Revascularization and optimization decision	
PCI	696 (20.6%)
CABG	39 (1.2%)
Deferral	2643 (78.2%)

Values are median [IQR] or n (%). CABG indicates coronary artery bypass graft; *FFR* fractional flow reserve, *iFR* instantaneous wave-free ratio, *LAD* left anterior descending artery, *LCX* left circumflex artery, *LM* left main coronary artery, *PCI* percutaneous coronary intervention, *P*_*d*_*/P*_*a*_ resting distal coronary to aortic pressure, *RCA* right coronary artery

**Fig. 1:**
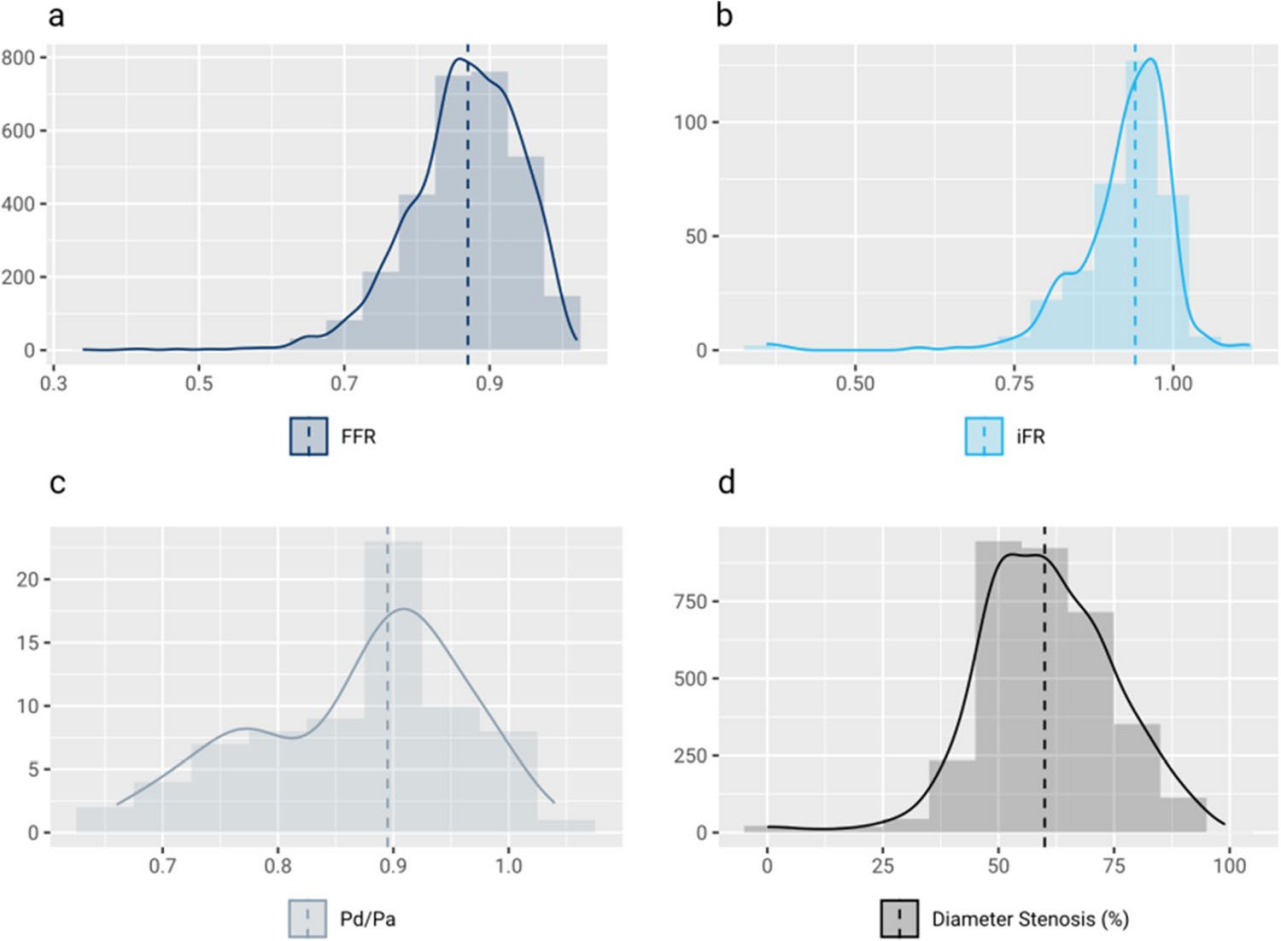
Distribution of pressure measurements and diameter stenosis. **a** Distribution of FFR measurement results. **b** Distribution of iFR measurement results. **c** Distribution of P_d_/P_a_ measurement results. **d** Distribution of diameter stenosis

Physiological assessment was performed in 71 lesions of the left main coronary artery (2.1% of all measurements), 1,964 lesions of the left anterior descending artery (58.1%), 745 lesions of the left circumflex artery (22.1%), and 598 lesions of the right coronary artery (17.7%). In 414 cases (12.3%), the pressure index was measured in a coronary side branch, and in another 10 cases (0.3%), the pressure index was measured to evaluate a bypass graft. In 125 (3.7%) cases, the pressure index was measured in the culprit lesion of an acute myocardial infarction. The median visually estimated diameter stenosis of all lesions was 60 (IQR: 50–70) % (Fig. 1d). When defining an “intermediate” coronary stenosis as any angiographic diameter stenosis of 40–90%, pressure index measurements were performed in an intermediate stenosis in 3,269 (96.8%) lesions.

**Fig. 2:**
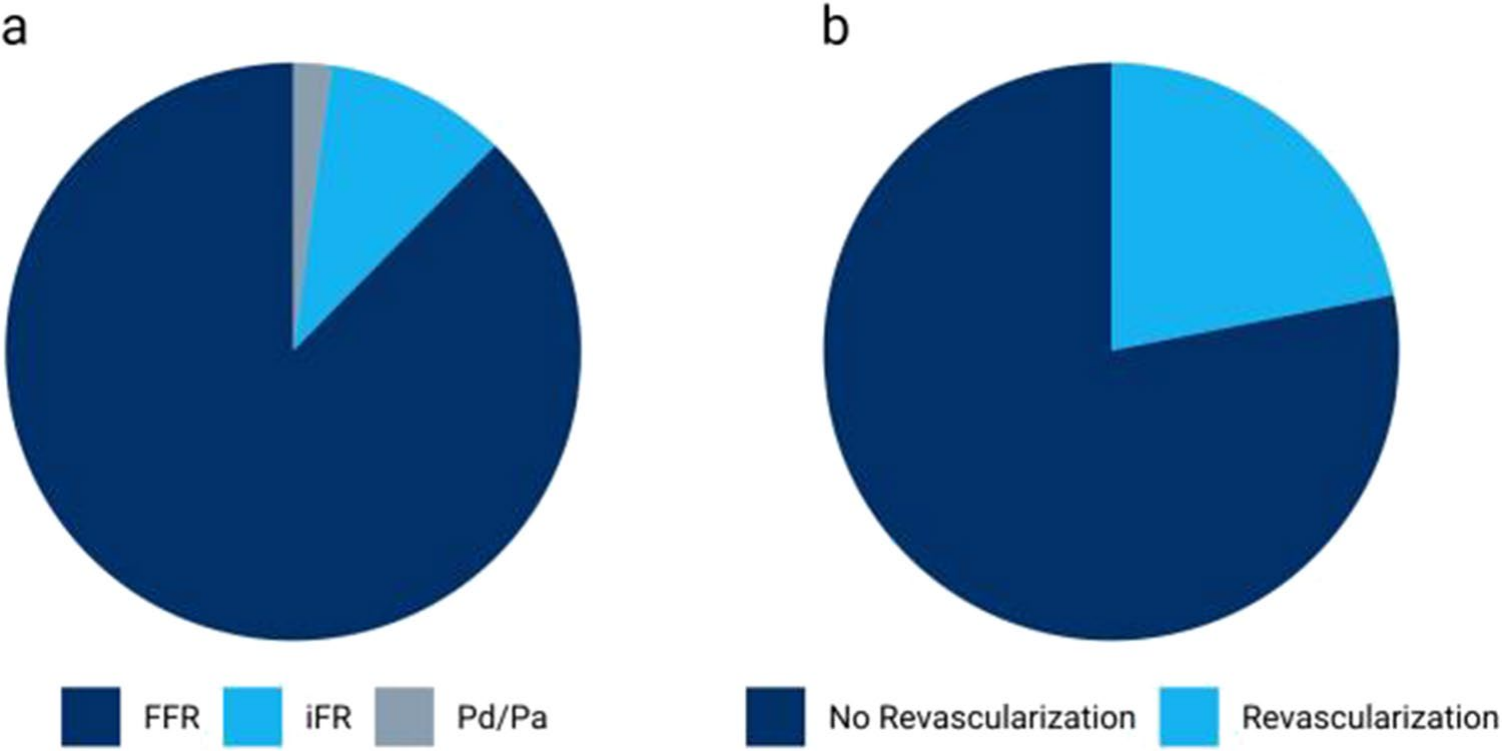
Use of coronary physiology in clinical practice. **a** Choice of intracoronary pressure index in clinical practice. **b** Revascularization decision after intracoronary pressure measurement for guidance of revascularization in clinical practice

FFR was used in 2,960 lesions (87.6%) and was therefore the most common pressure index (Fig. 2a). Hyperemia was induced by an intracoronary bolus of adenosine in 2,556 (86.4%) measurements and by an intravenous infusion of adenosine in 384 (13.0%) measurements. In 10 (0.3%) further measurements, vasodilation was achieved by an administration of regadenoson. The adenosine-independent pressure indices iFR and P_d_/P_a_ were obtained in 346 (10.2%) and 72 (2.1%) lesions, respectively.

The median FFR was 0.87 (IQR: 0.34–1.02), the median iFR, 0.92 (IQR: 0.36–1.12), and the median P_d_/P_a_, 0.87 (IQR: 0.66–1.04) (Fig. 1a-c). In the vast majority, i.e., 3,238 (95.9%) lesions, the pressure index was measured to guide revascularization decisions, while in 140 (4.1%) lesions the pressure index was measured to evaluate the result following PCI.

### Revascularization decisions

Following intracoronary pressure measurement to guide revascularization decisions, revascularization was deferred in 2,529 lesions (78.1%), whereas PCI was performed in 671 lesions (20.7%) and CABG was recommended in 38 cases (1.2%, Fig. 2b). The median visually estimated diameter stenosis of lesions that underwent revascularization following intracoronary pressure measurement was 70 (IQR: 60–80) % as compared to 50 (IQR: 50–70) % in lesions in which revascularization was deferred (*p* < 0.001). The median FFR and iFR of lesions which underwent revascularization were 0.77 (IQR: 0.74–0.79) and 0.84 (IQR: 0.81–0.88), respectively, while the median FFR and iFR of lesions in which revascularization was deferred were 0.89 (IQR: 0.85–0.93) and 0.96 (IQR: 0.93–0.98) (*p* < 0.001). If FFR or iFR values were below the threshold to recommend revascularization (≤ 0.80 for FFR and ≤ 0.89 for iFR, respectively), revascularization was performed in 93.7% of cases. In these lesions the median FFR was 0.76 (IQR: 0.72–0.78) and the median iFR was 0.83 (IQR: 0.81–0.87) if revascularization was performed accordingly and the median FFR was 0.77 (IQR: 0.73–0.79) and the median iFR was 0.87 (IQR: 0.85–0.89) if revascularization was deferred. Conservative treatment was chosen in 94.7% of lesions for which FFR or iFR values were above the revascularization threshold. Compared to a median FFR of 0.89 (IQR: 0.85–0.93) and iFR of 0.96 (IQR: 0.93–0.98) in lesions where revascularization was deferred accordingly, with a median FFR of 0.81 (IQR: 0.80–0.83) and iFR of 0.91 (IQR: 0.90–0.92) measured pressure indices were significantly lower (*p* < 0.001 for both FFR and iFR) in lesions where revascularization was performed despite coronary physiology demonstrating no hemodynamical relevance (Table 3). In total, the revascularization decision therefore agreed with the hemodynamic assessment in 94.5% of lesions.

**Table 3:** Revascularization Decision (*n* = 3,168 lesions)

	FFR ≤ 0.80 or iFR ≤ 0.89	FFR > 0.80 or iFR > 0.89
**Revascularization**	550 (93.7%)	138 (5.3%)
FFR	0.76 [0.72–0.78]	0.81 [0.80–0.83]
iFR	0.83 [0.81–0.87]	0.91 [0.90–0.92]
**No Revascularization**	37 (6.3%)	2,443 (94.7%)
FFR	0.77 [0.73–0.79]	0.89 [0.85–0.93]
iFR	0.87 [0.85–0.89]	0.96 [0.93–0.98]

Values are median [IQR] or n (%). FFR indicates fractional flow reserve; *iFR* instantaneous wave-free ratio

Among lesions in which intracoronary pressure indices were obtained to evaluate the physiological result after PCI, subsequent post-dilation or additional stent deployment was performed in 25 cases (17.9%) and subsequent referral to CABG occurred in one case (0.7%), whereas no further optimization was deemed necessary in the remaining 114 cases (81.4%).

### Intracoronary versus intravenous adenosine

**Fig. 3:**
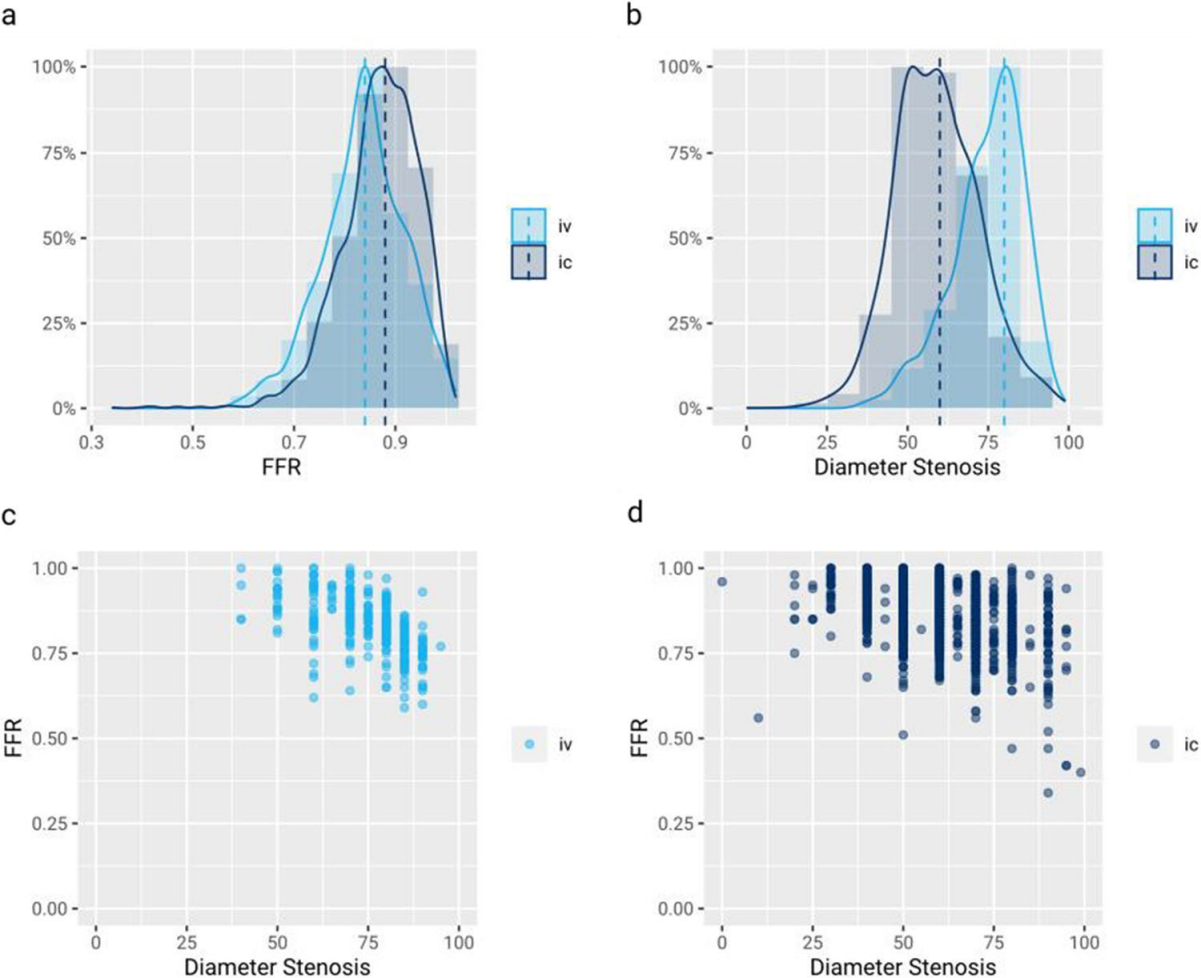
Intravenous versus intracoronary adenosine. **a** Relative distribution of FFR measurement results for intravenous and intracoronary administration of adenosine. **b** Relative distribution of visually estimated diameter stenosis for intravenous and intracoronary administration of adenosine. **c** Correlation of diameter stenosis with FFR for intravenously administered adenosine. **d** Correlation of diameter stenosis with FFR for intracoronary administered adenosine (iv = intravenous, ic = intracoronary)

In 3,238 lesions, intracoronary pressure measurements were performed prior to a revascularization decision and in 2,806 of these, adenosine was administered to measure FFR. In 381 of these 2,806 measurements (13.6%), adenosine was administered intravenously, while in 2,425 measurements (86.4%), adenosine was injected directly into the coronary artery at a dose of 40 to 800 µg. Median resulting FFR values were 0.84 (IQR: 0.79–0.89) for intravenous and 0.88 (IQR: 0.83–0.92) for intracoronary adenosine (*p* < 0.001) (Fig. 3a). However, the use of intravenous versus intracoronary administration of adenosine was heavily influenced by the clinical site, as was the visually estimated degree of stenosis of the lesions in which FFR was measured. The median diameter stenosis was 80 (IQR: 70–80) % for intravenous and 60 (IQR: 50–70) % for intracoronary adenosine (*p* < 0.001) (Fig. 3b-d). After correction for diameter stenosis, the route of adenosine administration was no longer an independent predictor of the resulting FFR value: it resulted in no more than a subtle change-in-estimate of -3.1% for a linear regression model to predict FFR from diameter stenosis. Agreement between the hemodynamic assessment and the subsequent revascularization decision was 93.4% for intravenous and 95.0% for intracoronary adenosine, hence not significantly different (p = 0.261).

### Complications

Complications of pressure index measurements were rare (Table 4). The pressure wire was reported to have caused coronary artery occlusion in two (0.1%) and coronary artery dissection in three (0.2%) patients. In one (0.1%) patient, FFR measurement resulted in a dissection of the left main coronary artery and was fatal. In the context of pressure index measurement, ventricular fibrillation was observed once (0.1%) in another patient. Side effects of adenosine administration were occasionally observed with prolonged chest pain in one (0.1%) patient and transient third-degree atrioventricular block or asystole in 22 (1.2%) patients during FFR measurement, but none of these cases required specific treatment.

**Table 4:** Complications

	FFR (*n* = 1,797)	iFR (*n* = 160)	P_d_/P_a_ (*n* = 43)	Overall (*n* = 2,000)
Prolonged chest pain	1 (0.1%)	0 (0%)	0 (0%)	**1 (0.1%)**
3^rd^ Degree AV block / asystole	22 (1.2%)	0 (0%)	0 (0%)	**22 (1.1%)**
Ventricular fibrillation	0 (0%)	1 (0.6%)	0 (0%)	**1 (0.1%)**
Coronary artery occlusion	2 (0.1%)	0 (0%)	0 (0%)	**2 (0.1%)**
Coronary artery dissection	3 (0.2%)	0 (0%)	0 (0%)	**3 (0.2%)**
Death	1 (0.1%)	0 (0%)	0 (0%)	**1 (0.1%)**

Values are n (%). 3^rd^ degree AV block indicates third-degree atrioventricular block; *FFR* fractional flow reserve, *iFR* instantaneous wave-free ratio, *P*_*d*_*/P*_*a*_ resting distal coronary to aortic pressure

## Discussion

### Main findings

This large, multicenter registry prospectively enrolled 2,000 patients with a total of 3,378 invasive pressure index measurements to analyze the use of coronary physiology in clinical practice, associated revascularization decisions, the rate of complications and potential systematic differences between intracoronary and intravenous administration of adenosine in the context of FFR measurements. In the vast majority of cases, physiological assessment was performed to guide revascularization in angiographically intermediate stenoses of patients presenting with CCS. The preferred pressure index was FFR. It was clearly shown that intracoronary bolus versus continuous intravenous administration of adenosine did not affect the result of FFR measurements, which strengthens the support for the intracoronary route of adenosine administration as a convenient alternative to the intravenous infusion which requires a more elaborate workflow. According to expectations, physiological assessment resulted in the deferral of revascularization in approximately 4 out of 5 lesions. While serious complications of intracoronary pressure measurement were rare, the rate of coronary injury by the pressure wire was not negligible.

### Use of coronary physiology

Current guidelines recommend the use of coronary physiology to guide the revascularization of angiographically intermediate stenoses in patients presenting with CCS [1–3]. Accordingly, over ¾ of patients, in whom an invasive pressure index was measured, presented with CCS in this registry, which is largely in agreement with previous registries [10–13]. In the vast majority of cases, pressure index measurements were performed in angiographically intermediate stenoses if “intermediate” is defined as the range from 40 to 90% diameter stenosis. This is in accordance with the European guidelines for myocardial revascularization [2]. It should be noted that the US guidelines for coronary artery revascularization only define the stenosis range between 40 and 69% as “angiographically intermediate” [1]. This was the case in just a bit over 60% of lesions of our registry. Similar observations have been made in the ERIS study [22].

If lesions are not physiologically relevant according to the FFR or iFR measurement, current guidelines recommend deferring revascularization in angiographically intermediate stenoses [1, 2]. This has been shown to avoid unnecessary revascularization and thereby its procedure-related complications [4, 5]. Similar to previous registries [10, 13], the use of coronary physiology resulted in the deferral of revascularization in close to 80% of lesions in this registry. In line with previous registries [11, 12], interventional cardiologists decided to proceed in accordance with pressure index measurements in about 95% of cases.

According to guideline recommendations, revascularization was deferred in 77.1% of lesions, including 58.8% of lesions with a visually estimated stenosis degree ≥ 70%. Unfortunately, this study includes no follow-up data regarding the downstream event rate relative to stenosis degree, or relative to the absence or presence of other high-risk lesion characteristics such as angiographic “haziness”. Particularly in consideration of the recently published PREVENT trial, which suggests a potential outcome benefit of revascularization in lesions with high-risk anatomic characteristics [23], such data would be interesting.

The specific rationale of interventionalists to proceed with revascularization in 5.3% of cases even though intracoronary pressure measurements were above the threshold for hemodynamic relevance was not captured as part of the study protocol and remains unknown. With a median FFR of 0.81 and a median iFR of 0.91 in these lesions, measured pressure indices were close to the recommended revascularization threshold and significantly lower than in lesions where revascularization was deferred in accordance with coronary physiology. Therefore, it can be assumed that such decisions were most likely made taking into consideration the clinical context, such as typical symptoms attributed to the coronary lesion. Potentially, the presence of perceived anatomic “high-risk” criteria may also have played a role.

With the results of the DEFINE-FLAIR and iFR-SWEDEHEART trials proving noninferiority of iFR to FFR [6, 7], the novel adenosine-independent pressure index is now equally recommended in current guidelines [1–3]. Nevertheless, the use of iFR made up only about 10% of pressure index measurements and the use of P_d_/P_a_ was negligibly low. In comparison, approximately 18% of pressure index measurements were iFR measurements in the SWEDEHEART registry [24]. However, relative acceptance of iFR as compared to FFR has become slightly uncertain, given that iFR was associated with an increased risk of death at 5 years as compared to FFR in the pooled analysis of the DEFINE-FLAIR and iFR-SWEDEHEART trials [8, 9].

In 15% of cases, physiological assessment was performed in patients presenting with ACS. Among patients presenting with ACS and multivessel coronary artery disease, physiological testing with FFR may be useful to guide revascularization in non-culprit lesions [25–28]. In culprit lesions, however, FFR measurement is not recommended in the acute setting as transient microvascular dysfunction prevents true vasodilation and therefore valid FFR measurement [29]. Accordingly, among patients with myocardial infarction most pressure index measurements were performed in non-culprit lesions even though a pressure index was also occasionally measured in culprit lesions.

Physiological assessment was not exclusively performed in native coronary arteries, but also in 10 coronary artery bypass grafts in this registry. FFR-guided revascularization has been shown to be feasible and provide better clinical outcomes than an angiography-guided revascularization in patients with intermediate stenoses of coronary artery bypass grafts [30].

While coronary physiology is commonly used to guide the decision to perform revascularization, the use of coronary physiology to evaluate the result after PCI is rather uncommon in clinical practice [22]. This was also the case in our registry: about 4% of the invasive pressure index measurements were performed after PCI, even less frequent than reported in the ERIS study [22]. A postinterventional optimization strategy guided by coronary physiology has shown a tendency to improve the physiological result of interventions [31], but its impact on clinical outcomes remains to be investigated. In fact, the ongoing DEFINE GPS (ClinicalTrials.gov identifier NCT04451044) and FFR-REACT (Dutch trial register identifier NL6523) [32] trials currently study the prognostic implications of the postinterventional use of coronary physiology.

Physiological mapping of coronary arteries by the pullback of a pressure wire allows discriminating between focal stenoses and diffuse coronary artery disease and may therefore facilitate identifying the optimal PCI target [33–36]. Despite the hemodynamic interaction between serial stenoses during hyperemia [37] limiting the value of FFR for pullback pressure registration, FFR was far more frequently used than iFR in this registry.

### Intracoronary versus intravenous adenosine

In our registry, intracoronary adenosine was used in the majority of cases. Intracoronary adenosine has been shown to induce hyperemia for FFR measurements equally well as a continuous intravenous infusion of adenosine [38]. The use of intracoronary adenosine is also supported by a recent meta-analysis, in which intracoronary adenosine demonstrated equivalent diagnostic accuracy, but was associated with less frequent side effects compared to intravenous adenosine [18]. Likewise, the route of adenosine administration did not affect FFR results in this registry either. We furthermore demonstrated that agreement between hemodynamic assessment and the subsequent revascularization decision did not differ significantly depending on the route of adenosine administration. Hence, our data further strengthens the evidence that the route of adenosine administration does not relevantly affect revascularization decisions in clinical practice.

### Complications

The use of adenosine-independent pressure indices obviates side effects caused by the administration of adenosine for hyperemia. Consequently, patients experience chest pain and dyspnea less frequently [6, 7]. Prolonged chest pain was only observed once during FFR measurement in this registry, but never with adenosine-independent pressure indices. Furthermore, adenosine sometimes causes arrhythmia, especially transient third-degree atrioventricular block [7, 13, 39, 40]. Likewise, third-degree atrioventricular block or asystole was reported in about 1% of FFR measurements, but not for adenosine-independent pressure indices in this registry.

Severe complications of invasive pressure index measurement were only rarely observed. In this registry, ventricular fibrillation occurred once during iFR measurement. However, ventricular fibrillation has been described previously in the context of the physiological assessment of coronary arteries [7, 39–41]. The pressure wire caused coronary artery dissection in 0.2% and coronary artery occlusion in 0.1% of our patients. Similar rates of coronary artery injury have been reported by previous registries and studies [10, 11, 13, 28, 39, 41].

Consequently, the rate of coronary artery injury is not negligible and should be borne in mind when considering invasive pressure index measurement. Coronary artery injury has even been reported to be fatal once in this registry and once in the Compare-Acute trial [28]. Clearly, a thoughtful use of coronary physiology to guide revascularization is required and interventional cardiologists should be aware of its potential complications.

### Study limitations

This registry reflects the use of coronary physiology in clinical practice in the participating 8 interventional cardiology centers. However, it seems likely the use of invasive pressure indices varies between interventional cardiology centers depending on preferences. Substantial heterogeneity was observed even between the participating centers and the registry is therefore not necessarily representative of the clinical use of coronary physiology in other centers.

The study protocol did not prescribe a specific algorithm to address the hemodynamic evaluation of serial lesions or lesions followed by a bifurcation, including left main coronary artery stenosis. For the latter, and particularly in left main bifurcation lesions, it is assumed that the investigators followed the recommended approach to measure pressure in both branch vessels as long as neither shows an angiographic lumen reduction [42, 43].

Novel methods for the evaluation of coronary physiology appear to be underrepresented in this study. The use of adenosine-independent pressure indices was relatively low while noninvasive, angiography-based pressure indices without the use of pressure wires or adenosine were not included in the registry.

The registry-based nature of this study obviously does not allow a randomized comparison between intracoronary and intravenous adenosine, hence making it subject to bias. Furthermore, the observative and nonrandomized design of a registry makes analysis, especially for correlation, vulnerable to confounding. Additionally, small systematic differences may have evaded detection as too few patients were included. While this study was able to compare intracoronary pressure measurement results, clinical decisions and complications following intracoronary or intravenous administration of adenosine, this study did not collect data on procedural duration and therefore is not able to analyze differences between the two approaches.

Since this registry primarily aimed to analyze the use of coronary physiology in clinical practice, no follow up was performed. Therefore, long-term clinical implications of the current practice regarding invasive pressure index measurement remain uncertain.

## Conclusions

In this large multicenter registry, intracoronary pressure measurements were mainly used to guide revascularization decisions in angiographically intermediate stenoses, which is in agreement with current guidelines. Importantly, this study provides further support that intracoronary administration of adenosine is a safe and equally effective alternative to the intravenous route of administration. The rate of pressure index-related coronary artery injury is low, but it is not negligible, so that careful decisions regarding the indication for wire-based intracoronary pressure measurements are required in every single case.

## Abbreviations

ACS: Acute coronary syndrome
CABG: Coronary artery bypass graft surgery
CCS: Chronic coronary syndrome
iFR: Instantaneous wave-free ratio
IQR: Interquartile ranges
FFR: Fractional flow reserve
F(FR)^2^: Fractional Flow Reserve Fax Registry
PCI: Percutaneous coronary intervention
